# P-439. Breast Cancer (BC) and Colorectal Cancer (CRC) Screenings among Persons with HIV (PWH): The Role of Individual and System-Level Factors on Screening Completion

**DOI:** 10.1093/ofid/ofae631.639

**Published:** 2025-01-29

**Authors:** Cole T Bredehoeft, Jing Peng, Ashley Lipps, Mohammad Mahdee Sobhanie, Carlos Malvestutto, Susan L Koletar, Yesha Patel

**Affiliations:** The Ohio State University Wexner Medical Center, Columbus, Ohio; The Ohio State University Wexner Medical Center, Columbus, Ohio; The Ohio State University Wexner Medical Center, Columbus, Ohio; The Ohio State University, Columbus, Ohio; The Ohio State University Wexner Medical Center, Columbus, Ohio; Ohio State University, Columbus, Ohio; The Ohio State University Wexner Medical Center, Columbus, Ohio

## Abstract

**Background:**

Non-AIDS-defining cancers (NADCs) are a leading cause of morbidity and mortality among PWH. With healthcare digitization and evolving HIV care models, equitable access to preventative care is crucial. We investigated individual and system-level factors associated with completion of BC and CRC screenings.

**Figure:**
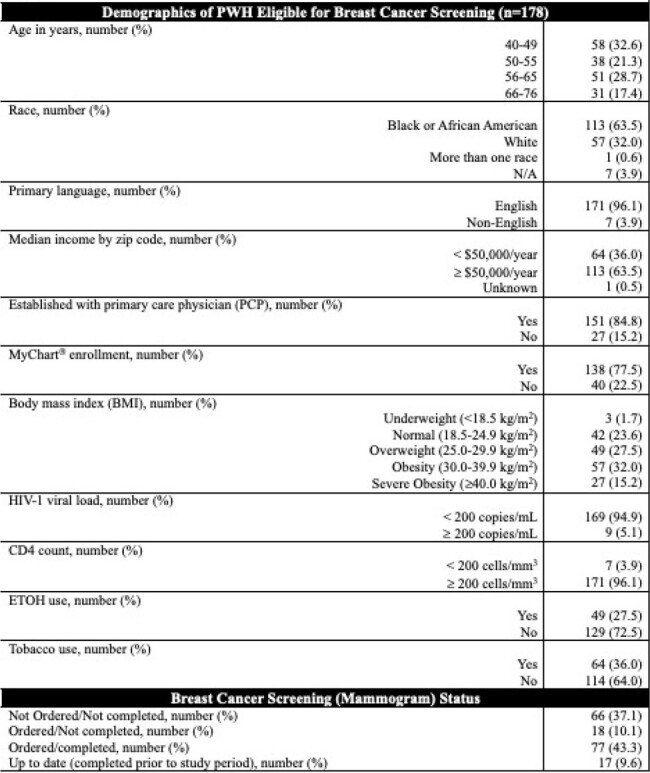

**Methods:**

This was a single-center, retrospective cohort study from 7/1/2022 to 7/1/2023. Established PWH (≥ 3 appointments between 7/1/2020-7/1/2023 with ≥ 1 within the study period) aged 40-75 were included. The primary outcome was ordering and completion of BC and CRC screenings. Demographics were summarized using frequency and percentage for groups with differing screening statuses. Chi-Square or Fisher’s exact test were used to evaluate associations between factors and outcomes. Descriptive statistics and p values were considered for evaluating associations between characteristics and screening status.

**Figure:**
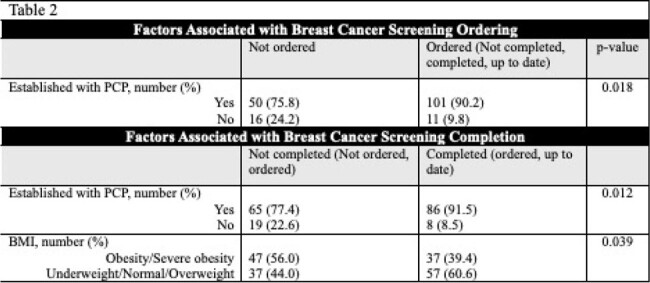

**Results:**

178 PWH eligible for BC screening were included. 113 (63.5%) were of Black/African American race, 151 (84.8%) had a primary care physician (PCP), and 138 (77.5%) were enrolled in an electronic medical record application (MyChart^®^). 52.7% were up to date on BC screening. Demographics and BC screening statuses are listed in Table 1. We observed a statistically significant association between having a PCP (90.2% vs 75.8%; p=0.018) and ordering of BC screening. Moreover, patients who completed BC screening are less likely to have obesity (39.4% vs 56.0%; p=0.039) (Table 2).

739 PWH eligible for CRC screening were included. 591 (80%) were assigned male sex at birth, 634 (85.8%) had a PCP, and 601 (81.3%) were enrolled in MyChart^®^. 349 (47.2%) were up to date on CRC screening. Demographics and CRC screening statuses are listed in Table 3. Significant associations were noted between having a PCP (90.8% vs 81.3%; p < 0.001), higher median income (79.6% vs 69.9%; p=0.003), and MyChart^®^ enrollment (88.0% vs 75.4%; p < 0.001) and CRC screening completion. Other notable variables are listed in Table 4.

**Figure:**
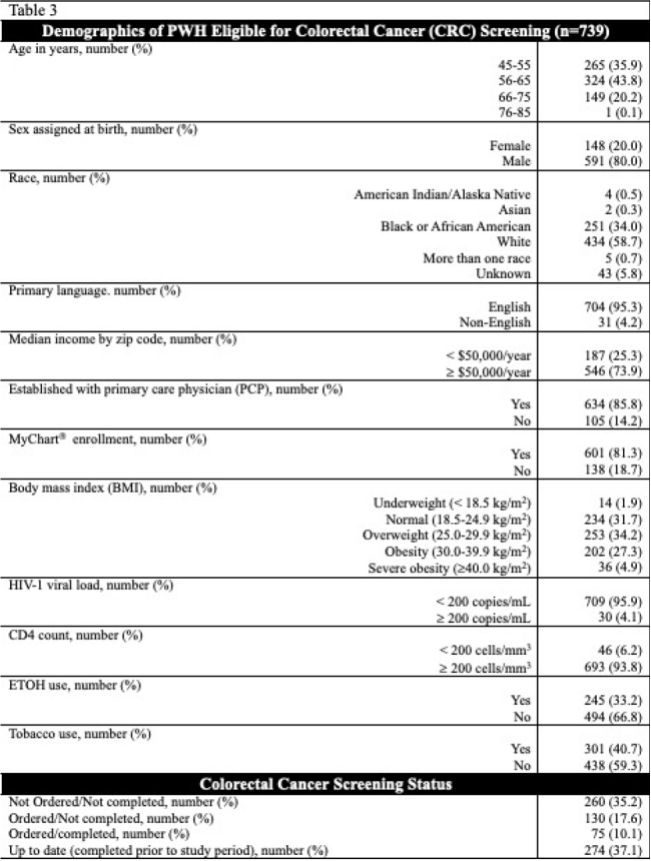

**Conclusion:**

BC and CRC screening is essential in reducing morbidity, mortality, and disparities of BC and CRC among PWH. Our findings provide insight on target populations for interventions and highlight opportunities to utilize digital technologies to improve cancer screening rates.

**Figure:**
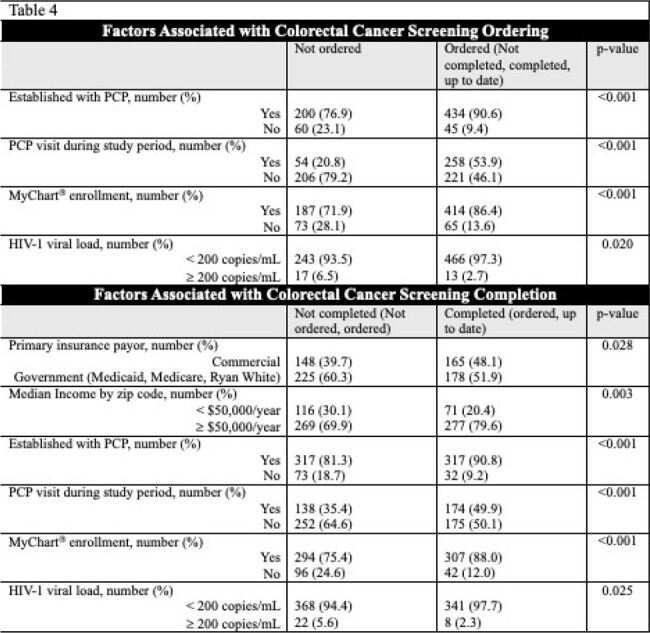

**Disclosures:**

**Carlos Malvestutto, MD MPH**, Gilead Sciences: Advisor/Consultant|Viiv Healhcare: Advisor/Consultant

